# Ventricular Subgaleal Shunt in Children Under Three Months of Age, from Diagnosis to Outcome: A Review After 11 Years of Experience in a French University Hospital

**DOI:** 10.3390/children12080983

**Published:** 2025-07-26

**Authors:** Timothée Follin-Arbelet, Alexandra Chadie, Jean-Baptiste Muller, Sophie Curey, Julien Grosjean, Cécile Toulemonde, Stéphane Marret

**Affiliations:** 1 Department of Pediatric Surgery, Rouen University Hospital, 37 Bd Gambetta, 76000 Rouen, France; 2 Department of Neonatal Pediatrics, Intensive Care and Neuropediatrics, Rouen University Hospital, 37 Bd Gambetta, 76000 Rouen, France; alexandra.chadie@chu-rouen.fr (A.C.); jean-baptiste.muller@chu-rouen.fr (J.-B.M.); stephane.marret@chu-rouen.fr (S.M.); 3 INSERM Unit 1245, Team 4, Rouen School of Medicine, Normandy University, 76000 Rouen, France; 4 Department of Neurosurgery, Rouen University Hospital, 37 Bd Gambetta, 76000 Rouen, France; 5 Department of Biomedical Informatics, Rouen University Hospital, and Analyse Intégrée Multimodale en Santé (AIMS) Lab, University of Rouen Normandy, 76000 Rouen, France

**Keywords:** hydrocephalus, ventricular subgaleal shunt, preterm infant, neurodevelopmental disorder

## Abstract

Background and objectives: Neurosurgical intervention on the newborn’s developing brain is a risk factor for neurodevelopmental disorders (NDDs). These patients necessarily require regular, coordinated follow-up. The ventricular subgaleal shunt (VSGS) technique has been used since 2013 at Rouen University Hospital. Like any change in practice, this technique must be evaluated. In this paper, we describe the population of patients with hydrocephalus treated by VSGS, the complications associated with the procedure, and the outcome of these patients at two and six years old. Methods: This study was an observational, descriptive, retrospective, single-center study. Children included were those less than three months old with hydrocephalus treated by VSGS at Rouen University Hospital from January 2013 to December 2023. Data were anonymized and collected using EDSaN software. A descriptive analysis was performed. Results: Thirty-two patients were included in our study. Of these, 22 (69%) were born prematurely; 16 (50%) of these 22 had postnatal intraventricular hemorrhage (IVH) requiring treatment with VSGS. A total of three patients (13.6%) died within the first year of life; twenty-four patients (75%) required definitive shunting. Twenty-two patients were over 2 years old in our study. Only 10 of them acquired the ability to walk (45%). Cerebral palsy was present in 10 (45%) patients. Fifteen patients were over 6 years old; thirteen (87%) attended school, but six (40%) had special needs (the need of an assistant, or part-time schedule). In our study, only 24 patients (82%) were followed by a pediatrician trained in neurodevelopment at Rouen University Hospital, and 27 (93%) were followed by a neurosurgeon. Conclusions: This study describes all patients with hydrocephalus treated by VSGS at Rouen University Hospital between January 2013 and December 2023, as well as their complications and their neurological outcomes. The follow-up of these children at risk of NDDs is essential.

## 1. Introduction

Hydrocephalus is an accumulation of cerebrospinal fluid (CSF) in the ventricles and pericerebral spaces. It is diagnosed by neuroimaging (transfontanellar ultrasound, magnetic resonance imaging (MRI) or brain computed tomography scan), which shows the dilatation of the cerebral ventricles [1]. It may be associated with high intracranial pressure (ICP). Clinical signs may appear. These include an increased head circumference (HC), bulging of the anterior fontanel, diastasis of the cranial sutures, projectile vomiting, altered general condition, lethargy, irritability, and sunset gaze. Signs of severity include bradypnea, bradycardia, coma, Kocher–Cushing triad (slow and irregular breathing, bradycardia, and elevated blood pressure), or even cerebral involvement [2].

Depending on the definition, neonatal hydrocephalus may be congenital if present at birth, primary if the cause is genetic (L1CAM), acquired or secondary if the cause is extrinsic (hemorrhage, tumor, infection), obstructive if there is an obstacle to the flow of CSF (stenosis of the aqueduct) or communicating if there is no obstacle, and syndromic if associated with other clinical and/or genetic anomalies (myelomeningocele) or isolated [3].

According to the Hydrocephalus Clinical Research Network, the main causes of neonatal hydrocephalus are, in order of frequency, intraventricular hemorrhage (IVH) of the preterm infant (26%), myelomeningocele (23%), obstructive hydrocephalus due to stenosis of the aqueduct of Sylvius (12%), cerebral or posterior fossa cysts (10%), congenital hydrocephalus (8%), post-infectious hydrocephalus (4%), and benign external hydrocephalus [1]. Appendix A shows an IVH on a coronal T2-weighted MRI slice.

Diuretic and subtractive treatments (lumbar punctions) have not been shown to be effective or safe and are not used [4,5,6]. Existing surgical treatments are either definitive (most commonly ventriculoperitoneal shunt) or temporary (external ventricular shunt, ventricular reservoir and subgaleal shunt). Various endoscopic treatments are also available.

Ventricular subgaleal shunt (VSGS) reduces pathologically elevated intracranial pressure by draining CSF under the galea. The main complications of VSGS are infections, malfunctions (obstructions, equipment displacement, and leaks), and hemorrhages [2,7]. Appendix B shows a VSGS on a coronal T2-weighted MRI slice.

For all these procedures, there is no consensus on the best time to intervene between the diagnosis of hydrocephalus and signs of severity. The etiology and evolution of hydrocephalus play an important role [8]. Concerning a specific population of preterm infants with IVH, in the ELVIS cohort (Early vs. Late Ventricular Intervention Study), there was no difference in early mortality and ventriculoperitoneal shunt (VPS) placement between short and long delays in infants with posthemorrhagic ventricular dilatation [9,10]. However, in a post hoc analysis, an earlier intervention was associated with a lower odds of death or severe neurodevelopmental disability in preterm infants with progressive posthemorrhagic ventricular dilatation. Leijser et al. also showed a better neurodevelopmental outcome in patients with an early approach to hydrocephalus management [11].

Brain development is complex and multi-factorial. It begins during pregnancy and continues into adulthood. Neurodevelopment enables the acquisition of motor, sensory, cognitive, behavioral, language, and social functions. This development is dynamic and responds to biological, genetic, epigenetic, environmental, and socio-cultural mechanisms [12]. The 2020 French Health Authority recommendations classify “major, prolonged and repeated surgery (cardiac, cerebral, abdominal, thoracic)” as risk factors for neurodevelopmental disorders (NDDs) [12]. The follow-up of patients undergoing surgery for VSGS placement must be multidisciplinary, in order to track down and appropriately manage these potential NDDs. NDDs include autism spectrum disorder (ASD), attention deficit disorder with or without hyperactivity (ADHD), intellectual development disorder, specific learning disorder, motor disorders, communication disorders, and other neurodevelopmental disorders (ICD11 and DSM-V). Cerebral palsy is one of the possible complications [13].

Most articles on hydrocephalus look through the prism of IVH in preterm infants. In 2018, Dr Gilard described the Rouen University Hospital’s experience of post-hemorrhagic hydrocephalus in 122 premature infants [14].

We chose to evaluate all newborns who benefited from the placement of a ventricular subgaleal shunt, since this technique was used routinely, between January 2013 and December 2023, i.e., over an eleven-year period.

The main objective of this study was to investigate and describe the general characteristics, complications, and outcomes of infants under 3 months old with hydrocephalus who needed a neurosurgical intervention consisting in a derivation with a VSGS at Rouen University Hospital, since this technique was first used there, over a period of 11 years.

## 2. Materials and Methods

### 2.1. General Characteristics of the Study

This is an observational, descriptive, retrospective, monocentric study conducted between 1 January 2013 and 31 December 2023 at the Rouen University Hospital, including all patients under three months of age who received a VSGS. All patients less than three months of age who had undergone at least one surgery with this type of device and who were hospitalized in the Neonatal Pediatric and Intensive Care or Pediatric Surgical Intensive Care departments of Rouen University Hospital were included. Patients who had not undergone VSGS were excluded.

### 2.2. Data Collection

Screening and data collection were extracted by EDSaN, the Rouen University Hospital’s Health Data Warehouse [15]. This is a “large database” that routinely collects clinical and biological data in the course of care at the University Hospital through various business software packages. This warehouse was authorized by the CNIL in October 2020 (decision DT-2020-007 https://www.legifrance.gouv.fr/cnil/id/CNILTEXT000044045332/). The EDSaN exploits textual clinical data (medical reports, end of hospitalization reports, etc.) using automatic natural language processing and knowledge engineering algorithms to minimize false positives and maximize true positives when searching using keywords and Boolean operators (AND, OR, NOT).

For this study, we used a query of medical texts produced by the neonatal department since 1997. The search equation was as follows: “sub galeal shunt”~3 OR “subgaleal shunt”~3 OR “sub galeal drain”~3 OR “subgaleal drain”~3. The use of syntax ~3 allowed us to search for similar expressions, with a maximum of 3 interspersed words. No other filters were applied. This query gave us the files eligible for our study.

EDSaN then provided anonymized access to all the documents in the patients’ medical files. By reading these records, we included or excluded patients from our study, according to our criteria.

In the event of missing data, we used the Rouen University Hospital’s medical software: ICCA©, CDP2©, or the imaging PACS©. Anonymized data were entered into an Excel© spreadsheet.

Data collected for identified patients were the following: first of all, clinical data, such as date of birth, sex, term of birth, birth measurements (weight, height, head circumference), and their respective term-adjusted percentiles, and mode of delivery (vaginal birth or caesarean section). Then, clinical signs of hydrocephalus and/or high ICP, such as axial hypotonia, peripheral hypotonia, peripheral hypertonia or tremor, vomiting, bulging of the anterior fontanel, head circumference enlargement, disjunction of skull bone sutures, abnormal contact (irritability, crying, failure to open the eyes), abnormal gaze (sunset gaze or strabismus), epilepsy, opisthotonos, bradypnea, bradycardia, and collateral scalp circulation. We collected the etiology of the hydrocephalus, classified in two categories according to the date of diagnosis: antenatal or postnatal. It was specified whether the hydrocephalus was syndromic or not. We collected data describing the treatment used: date of first neurosurgical procedure, actual age in days and corrected age in weeks of amenorrhea (WA), side of shunt, the need for postoperative CSF punctures in the subgaleal pouch, and associated medical treatments (diuretics or subtractive lumbar punction (LP)); the need of more than one surgery after 31 days and the reasons for them; time to revision and the total number of neurosurgeries, as well as the lifespan of the 1st VSGS and 2nd VSGS; the need of a definitive shunt as VPS, Endoscopic Third Ventriculostomies (ETVs), and the timing of these surgeries. Data about acute complications following the surgery (<31 days after surgery) were collected: death, CSF leakage through the scar, fever, meningitis, hemorrhage, comital seizures, early surgical revision, and parenchymal hernia. Finally, we were able to collect follow-up data for children whose follow-up took place at Rouen University Hospital. We noted whether a neurosurgeon and/or a pediatrician carried out the follow-up. In our study, we chose to look at patient’s neurodevelopmental outcomes at 2 and 6 years old. At age 2, we checked for the acquisition of walking and language (word association), diagnosis of cerebral palsy, presence of vision and hearing disorders, epilepsy, persistent hydrocephalus, and death. We defined persistent hydrocephalus as radiological hydrocephalus with the presence of ventriculomegaly on the 2-year follow-up scans. During the follow-up, the mention of “cerebral palsy” included the patient as having cerebral palsy, without considering the GMFCS (Gross Motor Function Classification System) grade. The GMFCS classification describes the level of cerebral palsy according to the child’s motor skills and age [16]. At age of 6, we checked again for the acquisition of walking, we ensured that children went to school, we specified if any school accommodations were needed (presence of an assistant, part-time schooling), and we gathered information about the need for rehabilitation (motor physiotherapy, speech therapy, psychomotricity, occupational therapy, etc.). Finally, we noted whether there was an associated diagnosis of NDDs.

### 2.3. Statistical Analysis

Statistical analyses were carried out using LibreOffice© software. A descriptive analysis of the population was carried out. Using spreadsheet functions, we calculated the following statistical indicators: mean, median, and quartile for quantitative variables, and numbers with their corresponding percentages for qualitative variables.

The authors declare no conflicts of interest. The Scientific and Ethical Committee of EDSaN has given a favorable opinion for this work.

## 3. Results

### 3.1. Study Population

**Figure 1:**
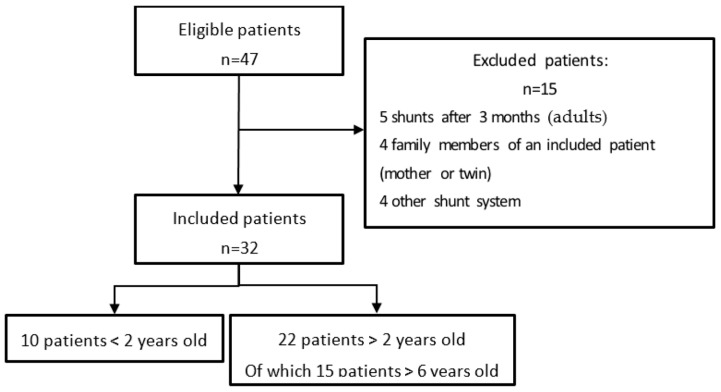
Flow chart summarizing the inclusion.

There were 47 patients eligible. Out of these 47 patients, 32 were included and 15 were excluded. The flow chart (Figure 1) summarizes the inclusion and exclusion process.

The population characteristics are summarized in Table 1.

Of all infants, preterm infants represented a significant proportion of our population, i.e., 69% (22/32) of the cohort.

**Figure 2:**
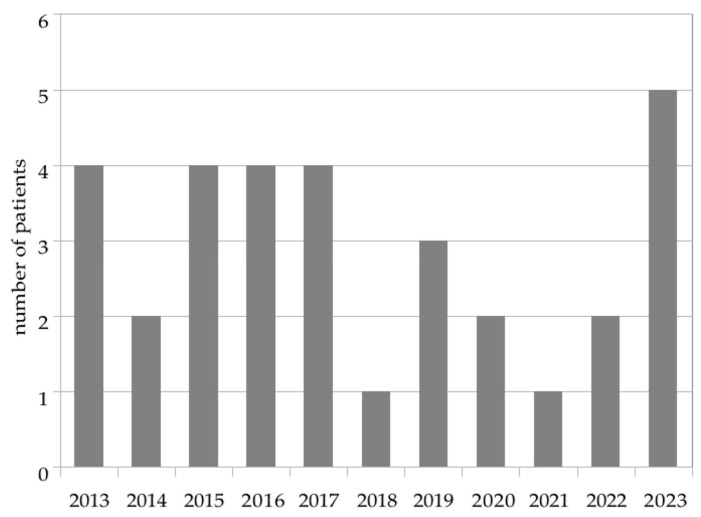
Population distribution year by year since the first VSGS in Rouen University Hospital.

The VSGS technique has been in use at Rouen University Hospital since 2013, with an average of 2.9 patients per year treated for the first time with a VSGS. The distribution is uneven: some years, only one patient benefited from a VSGS (2018, 2021) while for some other years, more than four children were treated with this technique (2023). Figure 2 represents this distribution.

### 3.2. Symptoms and Signs

**Figure 3:**
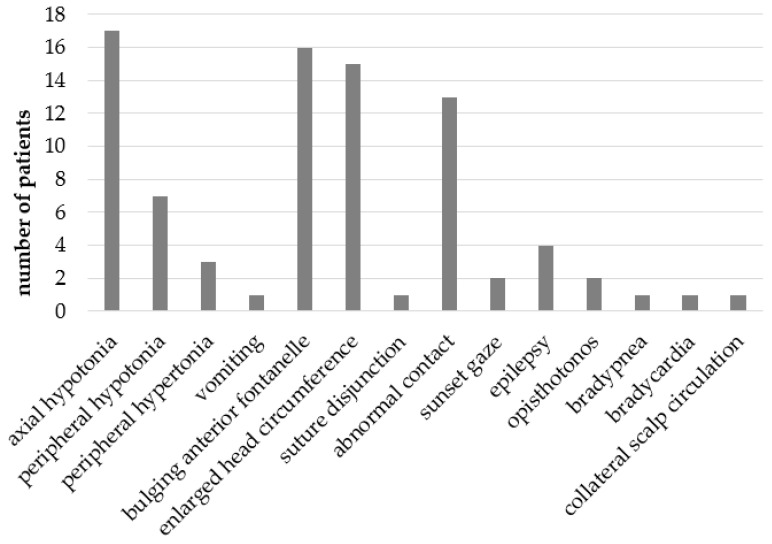
Clinical signs prior to the first VSGS and number of patients for each sign.

The most common symptoms were axial hypotonia in 17 patients (53%), a bulging of the anterior fontanel in 16 patients (50%), HC enlargement in 15 patients (47%) and abnormal eye contact in 13 patients (40%). Clinical signs of severity or cerebral distress such as opisthotonos, bradypnea, and bradycardia were, respectively, present in two patients (6%), one patient (3%) and one patient (3%). Bradycardia and bradypnea were present in the same patient. Figure 3 shows the number of patients per symptom. 

Radiological assessments were performed pre-operatively and post-operatively. Preoperatively, the median LVR was 0.54 and 0.41, respectively. 

### 3.3. Diagnosis and Etiology

In our study, we chose to differentiate between forms of hydrocephalus discovered antenatally or postnatally, without taking into account whether it was primary or secondary. In our study, 10 patients had an antenatal diagnosis and 22 a postnatal diagnosis. Only one patient had a syndromic form. This patient is one of ten whose hydrocephalus was diagnosed before birth. His syndrome combines stenosis of the aqueduct of Sylvius, microtia, and agenesis of the left auditory canal. To date, there has been no genetic research to explain this patient’s syndrome.

**Figure 4:**
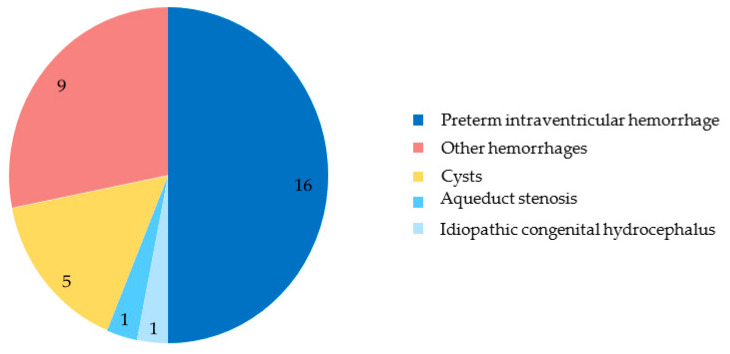
Number of patients according to etiology of hydrocephalus.

The etiologies identified in our patients fall into five categories: IVH of preterm infants, other hemorrhages, intracranial cysts, stenosis of the aqueduct of Sylvius, and idiopathic congenital hydrocephalus. IVH in preterm infants accounts for the largest proportion of our population, with 16 patients (50% of the population). Figure 4 summarizes the causes of hydrocephalus in our population.

Other hemorrhages accounted for nine patients (28%), including seven IVHs discovered before birth or in patients born at term, an ischemic and hemorrhagic stroke, and a cerebellar hemorrhage compressing the fourth ventricle. Totally, there are twenty-three patients with IVH (sixteen preterm infants and seven infants born at term): eleven (34%) had a grade 3 hemorrhage and twelve had a grade 4 (38%) hemorrhage according to the Papile classification. Cysts accounted for five patients (16%). These included three posterior fossa arachnoid cysts and two supratentorial cysts. The etiology of the last two patients was aqueduct stenosis (3%) and idiopathic congenital hydrocephalus (3%), respectively.

### 3.4. Treatment

The treatment characteristics are summarized in Table 2.

In our cohort, the median operative age was 19.5 days for real age and 36 weeks of gestation for corrected age.

All our patients underwent repeated surgeries. The median time between the first VSGS and second surgery was 27 days. The main reasons for the second surgery were as follows: revision of the first subgaleal shunt due to complications (obstruction, infection…) or ineffectiveness, placement of a second contralateral VSGS, or change to a permanent form of shunt (VPS or ETV). In the end, twenty-four patients (75%) had a permanent shunt: one had an ETV and twenty-three had a VPS. Of these last twenty-three patients, three had an ineffective ETV attempt, and therefore subsequently required a VPS. The median age at definitive shunt placement was 73 days.

### 3.5. Outcome

#### 3.5.1. Acute Complications

**Figure 5:**
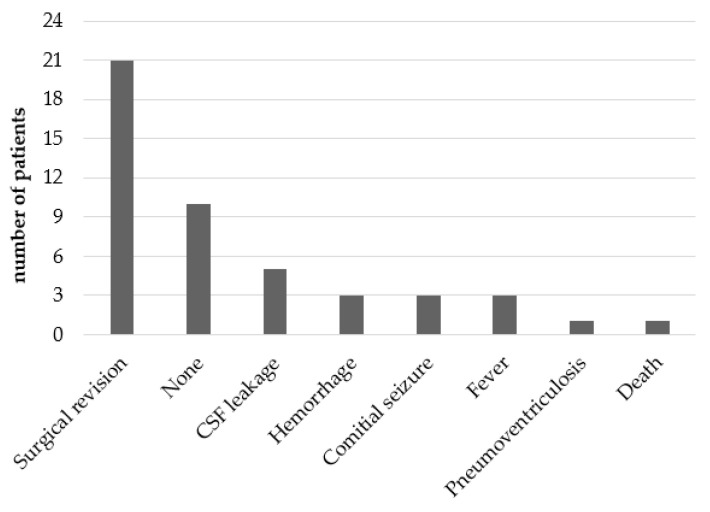
Acute complications after 1st shunt surgery.

Acute complications within 31 days of the first VSGS surgery are shown in Figure 5.

In our cohort, 21 patients (66%) underwent another surgery within 31 days of the first one. Ten patients (31%) had no acute complication. New surgery was always associated with re-hospitalization or transfer to the Intensive Care Unit. Three patients (9%) in our cohort developed fever, of whom two were diagnosed with meningitis (positive CSF culture). One patient (3%) died within the first 31 days post VSGS. Several post-operative complications occurred in the same patient.

#### 3.5.2. Neurological Outcome at 2 and 6 Years Old

In this cohort, 22 patients were aged over 2 years of age and 15 were over 6 years of age at the time of data collection. Of the total cohort, three patients (14%) died, including one within the first 31 days following the first VSGS surgery, and the others within the first year of life. These three deaths were due to post-operative complications involving cerebral hemorrhage, severe infection, and status epilepticus.

Of the entire cohort, at the time of data collection, 24 of the 29 surviving patients (82%) were followed by a hospital pediatrician trained in the diagnosis of NDDs or by a neuropediatrician, and 27 patients (93%) were followed by a neurosurgeon. Of the 22 patients over 2 years old, only 10 (45%) acquired the ability to walk and 11 (50%) had normal language abilities (word association). Hydrocephalus was still present in six patients (27%). Our cohort included 15 patients (47%) over 6 years old. At 6 years, 10 out of 15 patients (67%) were walking. Among them, four who were not walking at two years finally acquired the ability to walk. Of these fifteen patients who were 6 years old, thirteen (87%) were attending school, including six (40%) with special needs, i.e., receiving support from an assistant accompanying students with disabilities or requiring an adapted part-time schedule. The other two patients were cared for in a specialized institute for children with disabilities. Three of the six patients over 6 years of age had associated NDDs. These three patients have global acquisition delay, autism, and ADHD, respectively. Table 3 illustrates the outcome of patients aged between 2 and 6 years old.

## 4. Discussion

The goal of our retrospective, single-center study was to describe the population of infants under 3 months old with hydrocephalus, and treated neurosurgically with a VSGS, over an 11-year period between January 2013 and December 2023, to assess acute complications and evaluate the neurological outcomes of the children at ages 2 and 6.

In our cohort of 32 patients, 14 (43%) were female; 22 (69%) were born prematurely. The main etiology of hydrocephalus needing surgery was IVH in preterm infants, accounting for 50% of causes in sixteen patients; other hemorrhages (antenatal or term IVH, ischemo-hemorrhagic stroke, and cerebellar hemorrhage) occurred in nine patients (28%); the third etiology was intracerebral cysts in five patients (16%). The most frequent acute complications (occurring within 31 days of surgery) in our study included revision surgeries in 21 patients (66%) due to infection or malfunction of the shunting system. In 10 patients (31%), there was no acute complication. Of all patients, 24 (75%) needed a definitive shunt either by VPS or ETV. At the end of the inclusion period, twenty-two patients were over 2 years of age, of which fifteen were over 6 years old. At 2 years old, their follow-up showed that walking was acquired in 10 (45%) of them; vision and hearing were normal in 17 patients (77%) and 19 patients (86%), respectively. At 6 years old, thirteen (87%) attended school, including six (40%) with special needs (presence of an assistant or part-time schooling). There were three deaths (9%) among our cohort; all occurred within the first year of life.

Concerning the characteristics of the population, we can note a significant number of preterm infants, since the total number of premature babies is 22 (69%). The median term of birth in our cohort was 33.3 WA. In our study, of all preterm births, six out of twenty-two (27%) were caused by hydrocephalus itself. Indeed, as the ventriculomegaly detected antenatally worsened during follow-up, a decision was made to extract the baby prematurely. Except for one of these six patients, the decision to undergo a subgaleal shunt was taken rapidly, even antenatally. Then, the surgery was performed between the 2nd and 4th day of life. For the other 16/22 (73%) children, hydrocephalus was a complication of prematurity and intraventricular hemorrhage (IVH) occurred post-natally.

The most frequent clinical signs of high ICP in our patients were those specific to neonatal high ICP: bulging of the anterior fontanel and enlargement of the HC, as well as axial or peripheral tone disorders. These tone disorders were present in just over half of our patients (17; 53%). Axial hypotonia, peripheral hypotonia, and peripheral hypertonia may or may not occur in the same patient.

In the literature, Anurag et al. conducted a prospective observational study over 3 years (August 2017 to October 2020). During this period, 30 children were treated with a subgaleal shunt, with a median birth term of 31.2 WA, although the inclusion criteria did not exclude children born at term [17]. In the Anurag cohort, all patients presented with bulging or tense anterior fontanel [17]. This is consistent with our study. The incidence of antenatal IVH is not really known, but Huang et al. present their experience in a short review of four cases of antenatal IVH [18]. Of the four cases, only one was treated with a VPS on the third day of life, two died antenatally or immediately postnatally, and the last one was lost to follow-up before birth.

The leading cause is IVH in premature infants, and this accounts for 50% of children in our cohort. Congenital hydrocephalus and Sylvius aqueduct stenosis are present in our cohort, but only for 3% each. In the case of the stenosis of the aqueduct of Sylvius, the reason lies above all in the reference surgical technique, which is ETV, because the hydrocephalus is non-communicating [19].

Mancha et al. present a cohort of 66 patients over ten years old, to discuss outcomes and costs. This cohort included twenty-five patients (37.9%) with post-IVH hydrocephalus, eighteen (27.3%) with Chiari II malformation, six (9.1%) with intracranial cyst, four (7.5%) with idiopathic hydrocephalus, four (6%) with myelomeningocele, three (4.5%) with Dandy–Walker malformation, two (3%) with aqueduct stenosis, and three (4.5%) with another cause [20].

A small proportion of our patients received associated treatments. Only one patient (3%), born in 2013, received a diuretic treatment. This indicates the end of a practice that has shown no efficacy [4,5]. In comparison, in a single-center retrospective study of neonatal surgical hydrocephalus between 1993 and 2009 at Rouen University Hospital, Labarre reported 8.6% (five of fifty-eight) of patients treated with diuretics (acetazolamide). In our cohort, four patients were treated with subtractive LP in addition to their VSGS, respectively, in 2013, 2019, 2020, and 2023. In the literature, subtractive LP is not recommended, as it does not benefit mortality, definitive drain placement, or disability [6]. The 12 patients in the Garcia-Navarro cohort received VSGS as a first treatment. In addition to this treatment, six patients (50%) benefited from LP [21]. It is difficult to find studies in the literature on the evaluation of VSGS other than those describing the management of post-hemorrhagic hydrocephalus, especially in the preterm population.

The lifetime of our VSGS was 50 days for the first shunt, with extremes ranging from 1 to 149 days. When a second VSGS was required, its median lifespan was 29 more days. In their study, Frassanito et al. presented a cohort of 63 patients, 49 of whom received a post-IVH subgaleal shunt [22]. The average length of stay was 32.2 days. Frassanito et al. also compare different series including more than 10 patients affected by post-hemorrhagic hydrocephalus and managed by VSGS or a neuroendoscopic lavage. In these various cohorts, VSGS had an average lifespan of 44 to 140 days. The definitive derivation rate for our cohort is 75% of children, counting VPS and ETV, compared with 88.6% for Frassanito’s cohort, which does not count ETV. In his report, this rate varies from 70.6% to 100% [23]. This rate is only 62.5% in the subgaleal shunt cohort of Lam et al. [23]. All these studies discuss VSGS in the context of intraventricular hemorrhage, whereas we included other causes.

In our cohort, at the end of follow-up in December 2023, three patients (13.6%) had died due to post-operative complications. For one of them, death occurred within the first 31 days after the first shunt. The other two died before the end of their first year of life. In a meta-analysis, Badhiwala presents mortality rates ranging from 0% to 52%, with an average of 12.1% [7].

Follow-up by a pediatrician trained in NDDs or by a neuropediatrician concerned 24 out of 29 living patients, i.e., 82% of our cohort. It is possible that this rate is underestimated by the monocentric collection method and that this follow-up is effective in other centers. However, this rate is insufficient if we take into account that the 2020 French Health Authority recommendations on NDDs place “major, prolonged and repeated surgeries (cardiac, cerebral, abdominal, thoracic)” as a high-risk factor for NDDs, and “cerebral or cerebellar malformations of undetermined prognosis (cystic malformations of the posterior fossa)” as a moderate-risk factor for developing NDDs [24]. Trained professionals should care for all our patients. Follow-up by a neurosurgeon concerned 27 patients (93%). There are therefore two patients without a follow-up by a neurosurgeon. One of these patients does not have a definitive shunt nor hydrocephalus, but the second does have a VPS in place; he has a pediatric follow-up. Follow-up at two and six years old was not protocolized in our study, as it is a retrospective study, and the data retrieved differed from patient to patient, depending on the follow-up and the practitioners involved, while follow-up studies use standardized scales such as the Bayley scale [10,25]. We monitored gait disorders and cerebral palsy. Gait disorders may include the delayed acquisition of walking or cerebral palsy affecting the lower limbs. Overall, the 12 patients (55%) did not acquire the ability to walk at 2 years. Ten (45%) had a diagnosis of cerebral palsy (CP), testifying to the existence of visible or invisible brain and/or spinal cord injuries associated with HIV. We can compare our rates of CP with the ones in the study by Whitelaw et al. In Whitelaw et al.’s trial of the DRIFT method, which uses a tissue plasminogen activator injection into the ventricles associated or not with ventricular lavage, the inability to move without assistance affected 44% of patients treated by the DRIFT method and 52% by the standard method (Ommaya or Rickham-type VR) [25].

In our study population, 15 patients reached 6 years of age. Thirteen (87%) were attending schooling, while the other two (13%) were in a specialized institute for children with disabilities. Six of the fifteen patients (40%) had some form of special education (presence of an assistant, part-time schooling). Eleven patients (73%) followed rehabilitation programs such as physiotherapy, occupational therapy, speech therapy, or psychomotricity. Despite this significant need for educational or paramedical help, only three patients (20%) had an associated DSM-V diagnosis (ADHD, ASD...). In terms of comparison, we can nevertheless refer to the EPIPAGE 2 cohort, where help at school was required for 7% of premature babies born between 32 and 34 SA, 14% for those born between 27 and 31 SA, and 27% of premature babies born between 24 and 26 SA [26]. In this cohort, it appears that the greater the prematurity, the greater the need for help at school, whereas our cohort supports the fact that brain surgery is an important risk factor for NDDs.

In the specific case of the management of post-IVH hydrocephalus in preterm infants, the literature seems to answer one of the main outstanding questions: the delay in intervention. Depending on the degree of ventricular dilatation or the first signs of high ICP, surgical intervention is either “early” or “late”. Although this delay does not appear to modify acute complications or mortality, it does seem to have a better effect on the child’s neurodevelopmental trajectory when it is short [9,10,11]. In addition to this delay, some more recent surgical techniques such as neuroendoscopic lavage are still being evaluated. It was with this goal of standardizing practices while respecting patient safety and treatment efficacy that the TROPHY registry was created. This registry has been in operation since September 2018. It collects information prospectively, multicentrically, and internationally on newborns operated on before 41 SA for post-hemorrhagic hydrocephalus. It collects information on patient characteristics and the surgical procedure used, immediate postoperative complications, and outcomes at 6, 12, 24, 36, and 60 months [27].

### Strengths and Limitations

Our study focused on patients who were treated with a VSGS, making it an original work, since the majority of articles on this subject approach it through the prism of the cause of the hydrocephalus, mostly IVH of the preterm infants. Neonatal hydrocephalus is uncommon and severe, with 32 patients included, over a collection period of 11 years. Our cohort is similar in size to other studies on the subject, although there are articles in the literature with larger cohorts [17]. The use of the EDSaN database facilitated our research and enabled an exhaustive collection of records, in which the terms “subgaleal shunt” or “subgaleal drain” were found. No patient was lost to follow-up. This makes the results robust. The main limitations of our study are as follows: although the number of missing data are low, they are not entirely exhaustive. This classification bias is linked to the retrospective collection of data from computerized medical records for many variables, and therefore to a low level of scientific evidence. Furthermore, follow-up did not include a validated development scale, due to the retrospective method. At last, a monocentric study always has a selection bias due to the “center effect”, which is the case in our study. Non-protocol follow-ups result in a classification bias.

## 5. Conclusions

Hydrocephalus sometimes requires surgical management and the VSGS technique has demonstrated its efficacy and safety. Overall, research into neonatal hydrocephalus and its treatment is still in progress, with a need to standardize care for children. Locally at the University Hospital of Rouen, this retrospective work provides a humble insight into the subject, and sometimes points to a lack of follow-up for some patients. When neurosurgical intervention takes place in early childhood, the patient’s risk of developing NDDs increase. This is why medical follow-up must be rigorous, adapted, and coordinated over the long term. A pediatrician trained in NDDs and a neurosurgeon are essential for this follow-up. Depending on the child’s neurodevelopmental trajectory, other medical or paramedical professionals may also be required (physiotherapist, speech therapist, psychomotor therapist, etc.). It appears from our work that 15% of our patients have no appropriate pediatric follow-up, despite the increased risk of developing NDDs. Ensuring this long follow-up through collaborative work between pediatric and surgical teams is essential.

## Figures and Tables

**Table 1:** Population characteristics.

	General Population *n* = 32	MD
Gender, *n* (%) Female Male	14 (44) 18 (56)	
Term of birth in weeks of amenorrhea, median (IQ)	33.3 (28.5–36.8)	1
Preterm infant, n (%)	22 (69)	
Birth measurement, median (1st IQ–3rd IQ)		
Birth weight (g)	1960 (1285–2440)	1
Birth weight percentile	56 (24.5–78)	1
Birth height (cm)	43 (39–45)	3
Birth height percentile	49 (14–76)	3
Birth head circumference (cm)	31 (26.7–34.5)	1
Birth head circumference percentile	54.1 (17.3–90.25)	1
Delivery mode, *n* (%)		
Vaginal delivery Caesarean section	11 (34) 18 (56)	3

MD: missing data.

**Table 2:** Treatment characteristics of the 32 patients.

Age at first intervention, median (IQ) In days Age-adjusted (WA)	19.5 (13.0–34.5) 36.0 (33.5–38.6)
Shunt side, *n* (%) Right Left	23 (72%) 9 (28%)
Associated treatment, *n* (%) Diuretic Subtractive LP Subgaleal puncture	5 (15%) 1 (3.1%) 4 (12.5%) 2 (6.2%)
Surgical revision	32 (100%)
Cause of revision Infection 2nd shunt required 1st shunt revision Permanent shunt Hemorrhage	3 (12%) 8 (25%) 14 (44%) 5 (15%) 2 (6%)
Revision delay, in days, median (IQ)	27 (11–43)
Shunt life, in days, median (IQ) 1st ventriculosubgaleal shunt 2nd ventriculosubgaleal shunt	50 (29.2–76.2) 29 (25–31)
Permanent CSF diversion Ventriculoperitoneal shunt Endoscopic third ventriculostomy None	24 (75%) 23 (72%) 4 (12.5%) 8 (25%)
Age at permanent CSF diversion, median (IQ)	73 (57–98)
Total number of neurosurgical procedures, median (IQ)	4 (3–6.2)

WA: weeks of amenorrhea; LP: lumbar puncture; CSF: cerebrospinal fluid.

**Table 3:** Neurological outcomes at 2 years old and 6 years old.

	Population *n* = 32	MD
Follow-up by a pediatrician, n (%)	24 (75)	
Follow-up by a neurosurgeon, n (%)	27 (84)	
Death, n (%)	3 (13.6)	
Patients over 2 years of age, n (%)	n = 22	
Acquired walking	10 (45)	
Cerebral palsy	10 (45)	
Acquired language	11 (50)	1
Normal vision	17 (77)	
Normal hearing	19 (86)	3
Epilepsy	2 (9)	
Persistent Hydrocephalus	6 (27)	
Patients over 6 years of age, n (%)	n = 15	
Acquired walking	10 (67%)	1
Attending schooling	13 (87)	
Educational facilities	6 (40)	2
Rehabilitation	11 (73)	
Associated NDD	3 (20%)	1

MD: missing data; NDD: neurodevelopmental disorders.

**Figure A1:**
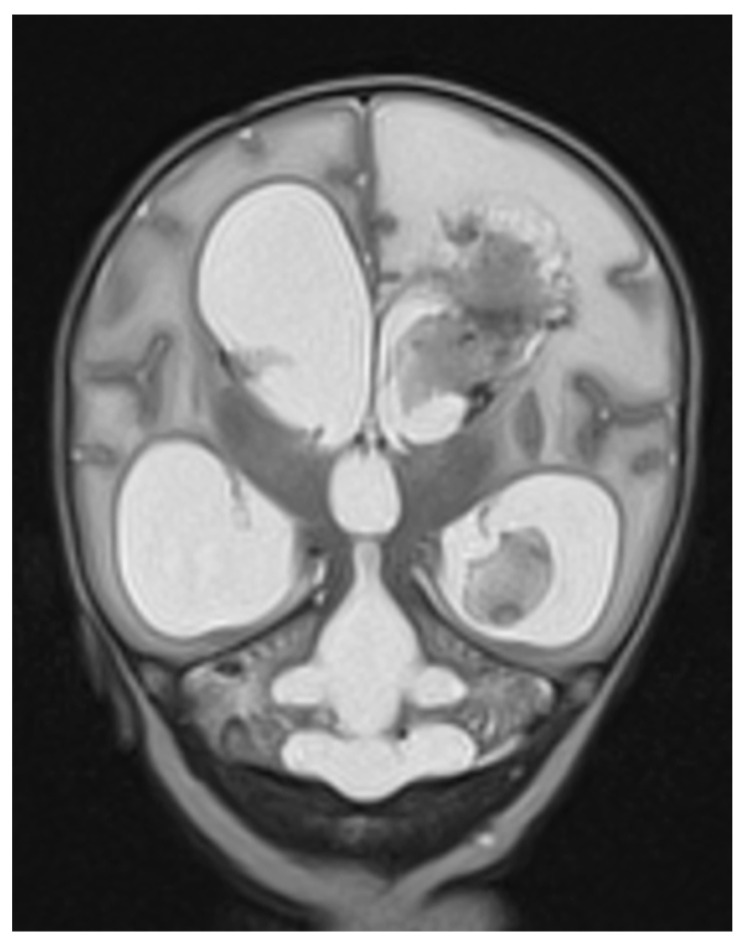
Left IVH with intra-parenchymal hemorrhage, MRI, T2 coronal section.

**Figure A2:**
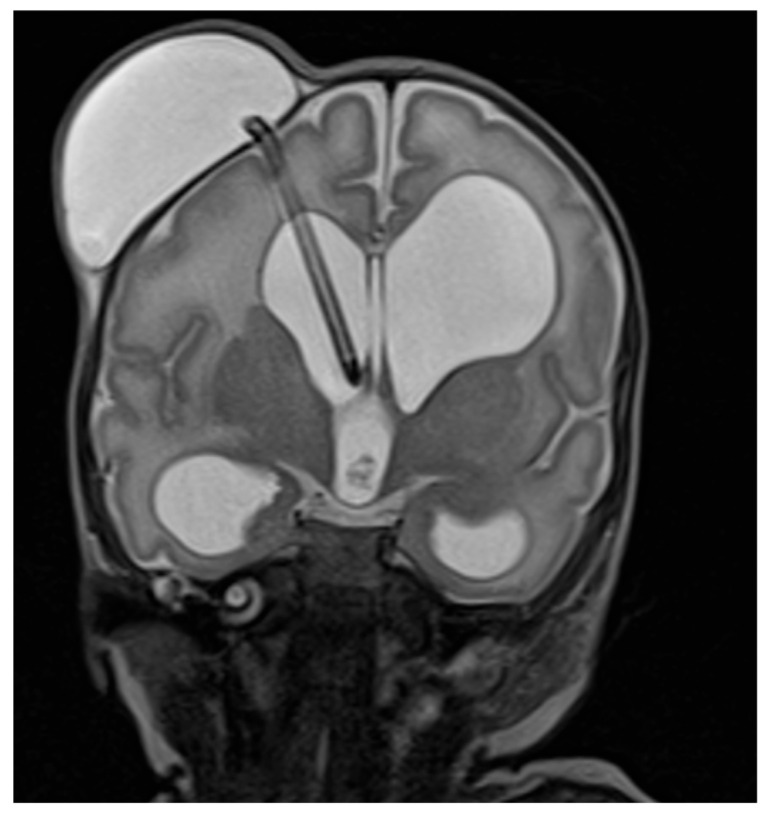
Right VSGS, drain visible between right lateral ventricle and right subgaleal pouch. MRI, T2 coronal section.

## Data Availability

No new data were created or analyzed in this study. Data sharing is not applicable to this article, because our study is a retrospective study.

## Abbreviations

The following abbreviations are used in this manuscript:

NDD	neurodevelopmental disorders
VSGS	ventricular subgaleal shunt
IVH	intraventricular hemorrhage
CSF	cerebrospinal fluid
MRI	magnetic resonance imaging
ICP	intracranial pressure
HC	head circumference
ELVIS	Early vs. Late Ventricular Intervention Study
VPS	ventriculoperitoneal shunt
ASD	autism spectrum disorder
ADHD	attention deficit disorder with or without hyperactivity
WA	weeks of amenorrhea
LP	lumbar punction
ETV	endoscopic third ventriculostomy
GMFCS	Gross Motor Function Classification System
IQ	inter quartile
CP	cerebral palsy

## References

[1] Flanders T.M., Billinghurst L., Flibotte J., Heuer G.G. (2018). Neonatal Hydrocephalus. NeoReviews.

[2] Pindrik J., Schulz L., Drapeau A. (2022). Diagnosis and Surgical Management of Neonatal Hydrocephalus. Semin. Pediatr. Neurol..

[3] Tully H.M., Dobyns W.B. (2014). Infantile Hydrocephalus: A Review of Epidemiology, Classification and Causes. Eur. J. Med. Genet..

[4] Whitelaw A., Brion L.P., Kennedy C.R., Odd D. (2001). Diuretic Therapy for Newborn Infants with Posthemorrhagic Ventricular Dilatation. Cochrane Database Syst. Rev..

[5] Kennedy C.R., Ayers S., Campbell M.J., Elbourne D., Hope P., Johnson A. (2001). Randomized, Controlled Trial of Acetazolamide and Furosemide in Posthemorrhagic Ventricular Dilation in Infancy: Follow-up at 1 Year. Pediatrics.

[6] Whitelaw A., Lee-Kelland R. (2017). Repeated Lumbar or Ventricular Punctures in Newborns with Intraventricular Haemorrhage. Cochrane Database Syst. Rev..

[7] Badhiwala J.H., Hong C.J., Nassiri F., Hong B.Y., Riva-Cambrin J., Kulkarni A.V. (2015). Treatment of Posthemorrhagic Ventricular Dilation in Preterm Infants: A Systematic Review and Meta-Analysis of Outcomes and Complications. J. Neurosurg. Pediatr..

[8] Levene M.I., Starte D.R. (1981). A Longitudinal Study of Post-Haemorrhagic Ventricular Dilatation in the Newborn. Arch. Dis. Child..

[9] de Vries L.S., Groenendaal F., Liem K.D., Heep A., Brouwer A.J., van ’t Verlaat E., Benavente-Fernández I., van Straaten H.L., van Wezel-Meijler G., Smit B.J. (2019). Treatment Thresholds for Intervention in Posthaemorrhagic Ventricular Dilation: A Randomised Controlled Trial. Arch. Dis. Child.-Fetal Neonatal Ed..

[10] Cizmeci M.N., Groenendaal F., Liem K.D., Van Haastert I.C., Benavente-Fernández I., Van Straaten H.L.M., Steggerda S., Smit B.J., Whitelaw A., Woerdeman P. (2020). Randomized Controlled Early versus Late Ventricular Intervention Study in Posthemorrhagic Ventricular Dilatation: Outcome at 2 Years. J. Pediatr..

[11] Leijser L.M., Miller S.P., van Wezel-Meijler G., Brouwer A.J., Traubici J., van Haastert I.C., Whyte H.E., Groenendaal F., Kulkarni A.V., Han K.S. (2018). Posthemorrhagic Ventricular Dilatation in Preterm Infants. Neurology.

[12] La Stratégie Nationale Autisme et Troubles du Neurodéveloppement (2018–2022)|handicap.gouv.fr. https://handicap.gouv.fr/la-strategie-nationale-autisme-et-troubles-du-neurodeveloppement-2018-2022.

[13] Surveillance of Cerebral Palsy in Europe (2000). Surveillance of Cerebral Palsy in Europe: A Collaboration of Cerebral Palsy Surveys and Registers. Surveillance of Cerebral Palsy in Europe (SCPE). Dev. Med. Child Neurol..

[14] Gilard V., Chadie A., Ferracci F.-X., Brasseur-Daudruy M., Proust F., Marret S., Curey S. (2018). Post Hemorrhagic Hydrocephalus and Neurodevelopmental Outcomes in a Context of Neonatal Intraventricular Hemorrhage: An Institutional Experience in 122 Preterm Children. BMC Pediatr..

[15] Pressat-Laffouilhère T., Balayé P., Dahamna B., Lelong R., Billey K., Darmoni S.J., Grosjean J. (2022). Evaluation of Doc’EDS: A French Semantic Search Tool to Query Health Documents from a Clinical Data Warehouse. BMC Med. Inform. Decis. Mak..

[16] Palisano R., Rosenbaum P., Walter S., Russell D., Wood E., Galuppi B. (1997). Development and reliability of a system to classify gross motor function in children with cerebral palsy. Dev. Med. Child Neurol..

[17] Anurag J., Sandeep C., Arunav S. (2023). Ventriculosubgaleal Shunt: An Institutional Experience. Childs Nerv. Syst. ChNS Off. J. Int. Soc. Pediatr. Neurosurg..

[18] Huang Y.-F., Chen W.-C., Tseng J.-J., Ho E.S.-C., Chou M.-M. (2006). Fetal Intracranial Hemorrhage (Fetal Stroke): Report of Four Antenatally Diagnosed Cases and Review of the Literature. Taiwan. J. Obstet. Gynecol..

[19] Cinalli G., Spennato P., Nastro A., Aliberti F., Trischitta V., Ruggiero C., Mirone G., Cianciulli E. (2011). Hydrocephalus in Aqueductal Stenosis. Childs Nerv. Syst. ChNS Off. J. Int. Soc. Pediatr. Neurosurg..

[20] Mancha G.T., Kadakia S., Muñoz L., Seske L.M. (2023). Ten-Year Review of Neonatal Neurosurgical Outcomes and Cost Analysis. Surg. Neurol. Int..

[21] Garcia-Navarro V., Perez-Vega C., Robles-Lomelín P., Valdez-Sandoval P., Vazquez P.M.G., Rodriguez Y.L., Cortes S.G.L., Naranjo E.C. (2021). Early Intervention and Neurodevelopmental Outcome of Infants with Posthemorrhagic Hydrocephalus: A Case Series and Literature Review. Clin. Neurol. Neurosurg..

[22] Frassanito P., Serrao F., Gallini F., Bianchi F., Massimi L., Vento G., Tamburrini G. (2021). Ventriculosubgaleal Shunt and Neuroendoscopic Lavage: Refining the Treatment Algorithm of Neonatal Post-Hemorrhagic Hydrocephalus. Childs Nerv. Syst..

[23] Lam H.P., Heilman C.B. (2009). Ventricular Access Device versus Ventriculosubgaleal Shunt in Post Hemorrhagic Hydrocephalus Associated with Prematurity. J. Matern. Fetal Neonatal Med..

[24] Troubles du Neurodéveloppement-Repérage et Orientation des Enfants à Risque. Haute Autorité de Santé. https://www.has-sante.fr/jcms/p_3161334/fr/troubles-du-neurodeveloppement-reperage-et-orientation-des-enfants-a-risque.

[25] Whitelaw A., Jary S., Kmita G., Wroblewska J., Musialik-Swietlinska E., Mandera M., Hunt L., Carter M., Pople I. (2010). Randomized Trial of Drainage, Irrigation and Fibrinolytic Therapy for Premature Infants with Posthemorrhagic Ventricular Dilatation: Developmental Outcome at 2 Years. Pediatrics.

[26] Pierrat V., Marchand-Martin L., Marret S., Arnaud C., Benhammou V., Cambonie G., Debillon T., Dufourg M.-N., Gire C., Goffinet F. (2021). Neurodevelopmental Outcomes at Age 5 among Children Born Preterm: EPIPAGE-2 Cohort Study. BMJ.

[27] Thomale U.-W., Cinalli G., Kulkarni A.V., Al-Hakim S., Roth J., Schaumann A., Bührer C., Cavalheiro S., Sgouros S., Constantini S. (2019). TROPHY Registry Study Design: A Prospective, International Multicenter Study for the Surgical Treatment of Posthemorrhagic Hydrocephalus in Neonates. Childs Nerv. Syst..

